# Climate change and women's health: Impacts and policy directions

**DOI:** 10.1371/journal.pmed.1002603

**Published:** 2018-07-10

**Authors:** Cecilia Sorensen, Virginia Murray, Jay Lemery, John Balbus

**Affiliations:** 1 University of Colorado School of Medicine, Department of Emergency Medicine, Aurora, Colorado, United States of America; 2 Public Health England, London, United Kingdom; 3 National Institute of Environmental Health Sciences, Bethesda, Maryland, United States of America

## Abstract

In a Policy Forum, Cecilia Sorensen and colleagues discuss the implications of climate change for women's health.

**Summary points**

- Climate change impacts on health—including increased exposures to heat, poor air quality, extreme weather events, altered vector-borne disease transmission, reduced water quality, and decreased food security—affect men and women differently, depending on local geographic and socioeconomic factors.
- Climate change threatens to widen existing gender-based health disparities, especially in low- and middle-income countries.
- Health impacts, and gender differences in those impacts, are mediated through socioeconomic, cultural, and physiologic factors. Policy action targeted towards these factors, which are often modifiable, can decrease negative health outcomes.
- Integration of a gendered perspective into existing climate, development, and disaster-risk reduction policy frameworks requires improvement in data acquisition, monitoring of gender-specific targets, coordination between sectors, and equitable stakeholder engagement.
- Empowering women as educators, caregivers, holders of knowledge, and agents of social change can improve mitigation and adaptation policy interventions.

## Introduction

As noted by the United Nations Framework Convention on Climate Change (UNFCCC) [1], women, especially those in poverty, face higher risks and experience a greater burden of climate change impacts. This is notably true for health impacts, making climate change a risk multiplier for gender-based health disparities. Both men and women are at risk for amplified health impacts. Women have distinct health needs, such as nutritional demands during pregnancy, which places them at risk of suffering from climate-sensitive diseases. Men experience other risks, such as suicide and severe depression in the face of drought [2] and resulting agricultural losses, and may be at higher risk of drowning during severe weather [3]. Compounding women’s health vulnerabilities are cultural constructs, which amplify risk on a regional scale. Globally, a total of 1.3 billion people in low- and middle-income countries live below the poverty line, and 70% are female [4]. Yet while the interactions between poverty, gender-based social discrimination, and climate change threaten to amplify gender-based health disparities, women’s social roles and potential for agency afford opportunities for promoting solutions to sustainability, disaster risk reduction, and solutions to health threats. Ensuring that policies move beyond traditional separations of health, gender, and environment and embrace proactive and gender-based solutions is paramount to protecting women’s health and mobilizing their vast social potential to mitigate, adapt to, and respond to climate threats.

## Health impacts

Climate change affects health through a multitude of mechanisms, including heat, poor air quality, and extreme weather events, as well as through meteorological changes that alter vector-borne disease, reduce water quality, and decrease food security [5]. The health risks associated with these exposure pathways are mediated through physiologic, cultural, and socioeconomic vulnerabilities, which differ substantially between men and women (Table 1). The lack of gender-disaggregated health data restricts conclusive understanding of thresholds of exposure for harm and may result in a lack of awareness by local, national, and even global decision makers and healthcare personnel. Many examples exist, including the following:

- Globally, women suffer from higher rates of anemia and malnutrition and are sensitive to climate-driven food insecurity due to increased nutritional needs during menstruation and childbirth. Anemia is associated with cognitive impairments including poor attention span, diminished working memory, and poor educational outcomes [6]. Additionally, a majority of the world’s smallholder farmers are women, and therefore women’s livelihoods are at risk from climate-related crop failure, which threatens to increase poverty as well as poor health outcomes.
- Respiratory and cardiovascular disease secondary to exposure to poor air quality preferentially impacts women due to a greater proclivity for higher deposition of particulate matter in lung tissue and higher rates of anemia [7]. Poor air quality is also associated with negative birth outcomes [8] and affects maternal/child health in that it is associated with stillbirth, intrauterine growth restriction, and congenital defects [9]_._ Women spend greater amounts of time in the home and thus are disproportionately exposed to particulate matter from the use of traditional indoor stoves for cooking and heating [10].
- During climate-related disasters, women suffer disproportionate mortality [10], and survivors experience decreased life expectancy [11].
- Women and girls are at a higher risk of physical, sexual, and domestic violence in the aftermath of disasters [12] and are at a higher risk for mood disorders and poor economic recovery [13]. These impacts are amplified when women have a lower socioeconomic status [4].
- Forced migration and repeated short-distance moves are especially significant for poorer people, as well as for groups such as women, who are often excluded from migration analysis [13].

**Table 1:** Examples of disparate climate-related health impacts on women and relevant physiologic, cultural, and socioeconomic risk factors.

Exposure pathway	Gender disparities in health impacts	Physiologic and biologic vulnerabilities	Cultural and socioeconomic vulnerabilities
Increasing frequency of extreme heat events and rising average seasonal temperatures	• Disproportionate heat-related morbidity and mortality	• Women have a higher working metabolic rate, reduced heat dissipation through sweating, and decreased effective radiative cooling [14]	• Poor access to healthcare and cooling facilities due to personal safety concerns and lack of access to personal transportation • Lack of communication and awareness of women’s vulnerabilities to heat among local, national, and even global decision makers and healthcare personnel • Dearth of gender-disaggregated heat-related health data, unknown critical exposure windows • Culturally prescribed heavy clothing garments
	• Adverse reproductive outcomes: preterm delivery [15], congenital defects [16], gestational hypertension, and pre-eclampsia [17]	• Heat increases production of vasoactive substances, increases blood viscosity, and affects endothelial cell function, which may alter placental blood flow and increase propensity for hypertensive crises and stillbirth [18] • Hyperthermia is teratogenic, disrupting the normal sequence of gene activity during organogenesis [16]	
Poor air quality from combustion of fossil fuels; increased ground-level O_3_ from elevated temperatures	• Respiratory and cardiovascular disease [19]	• Women experience greater deposition of inhaled particles in their lungs [19] • Secondary to higher prevalence of anemia, women are more sensitive to toxicological exposure [7]	• Traditional indoor stoves for cooking and heating utilize biomass, which produces carbon monoxide, hydrocarbons, and particulate matter and accounts for nearly 24% of ambient air pollution from PM_2.5_ [9]; women spend more time in the home and thus are disproportionately affected
	• Adverse reproductive outcomes: stillbirth, intrauterine growth restriction, and congenital defects [8,20,21]	• Air pollutants (e.g., CO_2_) can cross the placenta and impact fetal growth during crucial developmental windows • Air pollutants impair maternal respiratory and cardiovascular health and result in reduced efficiency of placental function with consequent deterioration in fetal development	
Increasing frequency of climate-related disasters, including hurricanes, flooding, and wildfires [22]	• Women suffer disproportionate mortality during natural disasters [15] • Female survivors suffer decreased life expectancy [16] • Women and girls are at high risk of physical and sexual violence, especially those belonging to marginalized sectors of society [12,23] • Women are at higher risk for mood disorders such as depression and anxiety after disasters [13] • Women giving birth in the time period following disasters have an increased risk of complications, including preeclampsia, bleeding, and low-birthweight infants [24]	• Women of all ages are more likely to experience dietary deficiencies, leading to poor physical health and vulnerability to resource shortages ensuing from catastrophes [25] • Poor baseline nutritional status and physical health may prevent escape and survival in the acute phase of disaster [26–28]	• Women have unequal access to basic social goods, and mortality is worsened when women have a lower socioeconomic status [10,29] • Women are often homebound caring for children and elderly while waiting for relatives to return prior to evacuation • Poor, single, elderly women, adolescent girls, and women with disabilities are often at greatest risk for abuse because they have fewer personal, family, economic, and educational resources from which to draw protection, assistance, and support • Women suffer disproportionate job loss and stagnant personal economic recovery following disasters [30] • Poor access to obstetric care during and after disasters
Shifting rainfall and temperature patterns impair crop, livestock, and fishery yields, contributing to food insecurity	• Women suffer higher rates of macro- and micronutrient deficiencies • Women suffer higher rates of anemia, which is associated with cognitive impairments including poor attention span, diminished working memory, emotional regulatory issues, and impaired sensory perception [6] • Malnutrition causes negative effects on neonatal outcomes including intrauterine growth restriction and perinatal mortality [31]	• Women are inherently sensitive to the effects of food insecurity and resulting nutritional deficiencies due to increased needs during menstruation, pregnancy, and nursing	• Nutritional scarcity can be intensified by cultural practices that prioritize food provision to children and adult males • In low-income countries, women produce between 60%–80% of all food; livelihoods, as well as nutritional status, are threatened when climatic conditions negatively impact agricultural yields [32] • Less than 10% of female farmers are landowners, and barely 2% have proper paperwork for their land [32], therefore women suffer on account of their relative lack of control over farmlands and nutritional security
Shifting rainfall and increased rates of evaporation lead to water insecurity and risk of waterborne disease	• Water scarcity forces provision from sources that may be biologically and toxicologically contaminated, resulting in bacterial, viral, and protozoan infections as well as toxin exposure [14] • Traveling long distances to procure water increases exposure to heat [33] • Lack of access to water and sanitation creates unsafe conditions for women, especially during reproductive times [34]	• Dehydration in pregnancy results in decreased uterine blood flow and is associated with preterm labor [15] • Infection in pregnancy leads to poor maternal and neonatal outcomes	• Traditionally, women have the household role of providing water for the family; water scarcity equates to more time spent harvesting water and less time spent on other activities of livelihood such as economic gain • In some regions, carrying water may use up to 85% of a woman’s daily energy intake [14] • Traveling long distances to collect water places women at risk for physical abuse and harm
Changes in temperature, precipitation, and ecology are altering the geographic distribution of vector-borne diseases	• Exposure to mosquito-borne illnesses poses health threats to pregnant women who are exceptionally vulnerable • Pregnant women have a risk of severe malaria that is 3 times as high as that of nonpregnant women [35] • Zika virus carries devastating fetal impacts, including microcephaly, CNS malformations, and impaired cognitive development [36] • Dengue virus is associated with increased risk of cesarean delivery, eclampsia, and growth restriction [37]	• Pregnant women have increased susceptibility to mosquito-transmitted diseases due to higher CO_2_ production, a chemoattractant for mosquitos, and increased peripheral blood flow, the heat from which allows mosquitos to locate hosts • Hormonally induced changes in immunologic function during pregnancy lead to decreased immune response, which manifests as higher intensity of viremia and parasitemia [38,39] • Infection during pregnancy can result in anemia and diminished transplacental nutrient transport resulting in intrauterine growth restriction and increased vulnerability of the mother to hemorrhagic complications of delivery [40]	• Women spend more time around the house performing domestic tasks, which places them in close proximity to domestic standing-water and mosquito-breeding sites • Lack of access to prenatal obstetric care and assisted deliveries places women with infections at risk of postpartum hemorrhage and poor maternal outcomes, including death
Climate-induced environmental change drives human migration and/or results in “trapped” populations	• Women are more likely to undergo short-term migration (versus long-distance migration), which is often excluded from migration analysis [41] • Women are more likely to displaced by drought [41]	• Lack of basic sanitation and health services compound health issues for refugees and migrants [42] • Forced migration as a result of environmental changes or disasters is physiologically and mentally stressful, leading to poor health outcomes [42] • Women whose partners travel frequently are at higher risk of HIV infection [42]	• Marriage is a key driver of internal migration for women • Low education is a risk factor for migration, and globally, women suffer disproportionately from lack of education • Women have fewer employment opportunities and therefore are unable to migrate into economically viable and less environmentally vulnerable regions, thus become “trapped” • Women who do migrate are still faced with the direct pressure of caring for children [42] • Migration places women at risk for human trafficking [42]

Abbreviations: CNS, central nervous system; PM_2.5_, inhalable airborne particles with diameters that are 2.5 micrometers and smaller.

## Policy frameworks

Climate change, poverty, and gender inequality are increasingly recognized as global problems; however, achieving the integration of policies, surveillance, and program creation and implementation necessary to make progress in solving these interrelated issues has proven challenging. For example, the Sustainable Development Goals (SDGs) contain separate targets for poverty (SDG 1), gender equality (SDG 5), sustainability (SDG 11), and climate action (SDG 13). Opportunities to interconnect these separate targets through subtargets and indicators that bridge sectors were largely lost during the development of the SDGs. Thus, while there are energy-related indicators in the health goal (related to household use of biomass fuels), there are no health-related indicators in the energy or climate goals. Disaggregation and failure to explicitly link health with these other goal areas leads to discordant efforts, inefficiencies, and communication barriers between involved agencies tasked with solving these multisectoral problems.

Similarly, some advances have been made within the UNFCCC and the United Nations International Strategy for Disaster Reduction (UNISDR). UNFCCC decision 21/CP.22 (2017) calls for a “gender action plan” to incorporate a gendered perspective in all elements of mitigation, adaptation, capacity, technology, and finance. Although this framework sets the stage for action, systematic integrative procedures are lacking, as are indicators to monitor progress. The Sendai Framework—an international covenant adopted in 2015 to establish common goals and standards for disaster risk reduction—formalizes climate change as a disaster-risk multiplier to women and recognizes women as important stakeholders in risk reduction [43]. Furthermore, it calls on adopters to “prepare, review and periodically update preparedness policies, plans and programmes with the involvement of all relevant institutions, considering climate change scenarios and their impact, and to facilitate the participation of all sectors and stakeholders” [43]. Strong accountability is fundamental to the framework, which contains 38 indicators to track progress in implementing the 7 targets, which aim to reduce disaster mortality and damage to critical infrastructure and economies through increased multihazard early-warning systems, improved national and local mitigation strategies, and enhanced international cooperation. The framework also incorporates the related dimensions of SDGs related to poverty, sustainability, and climate action. The Sendai Framework Monitor will also function as a management tool to help countries develop disaster risk reduction strategies, make risk-informed policy decisions, and allocate resources to prevent new disaster risks. It will also target disaggregated data collection with formal biannual reporting via the Sendai Framework Monitoring Process.

## Towards integration

Gender mainstreaming throughout all climate targets has been recognized by the UNFCCC as critical to increasing effectiveness. Women play a vital role in the societal response to climate change; their participation at all levels has been shown to result in greater responsiveness to citizens’ needs and often increases in cooperation across party and ethnic lines, generally resulting in sustainable outcomes [23,44]. In order to support this action, we propose that effective engagement and communication with women and girls throughout society must be included at all levels and within the following practices to support an integrative policy approach (Table 2).

**Table 2:** Examples of multisectoral solutions to climate change impacts on women’s health.

Exposure pathway	Impact on women	Gender-based solutions	Sectors involved
Increasing frequency of extreme heat events and rising average seasonal temperatures	• Increased morbidity and mortality and poor birth outcomes	• Provide air conditioning in maternal wards (shown to decrease intensive care need in neonatal period) [45] • Increase access to prenatal care in heat-vulnerable geographic areas • Implement heat early-warning systems with educational messages targeted at women • Collect and disseminate gender-disaggregated public health data • Consideration of the detrimental effects of urban heat islands, especially in regions with poor access	• Public health • Urban planning • Medicine
Poor air quality from combustion of fossil fuels; increased ground-level O_3_ from elevated temperatures	• Women spend a disproportionate amount of time in the home and thus are disproportionately affected by indoor air pollution, resulting in respiratory and cardiovascular disease	• Improve access to clean-burning cook stoves—shown to reduce exposure to carbon monoxide, hydrocarbons, and particulate matter and decrease health risks [46] • Consider women’s transportation needs during urban planning • Consider the impacts of poor air quality on fetal and maternal health and strive to reach PM_2.5_ targets in rural and urban environments [15]	• Transportation • Technology • Public health
Increasing frequency of climate-related disasters, including hurricanes, flooding, and wildfires	• Women suffer disproportionate mortality during disasters and are at high risk of abuse and poor economic and mental health recovery	• Provide gender-sensitive emergency shelters that proactively safeguard women • Provide emergency obstetric and gynecologic care very early in the course of disasters • Increase availability of gender-disaggregated disaster-related health data • Increase gender-specific public health messaging before, during, and after disasters • Provide gender-sensitive psychologic services in the aftermath of disasters • Create economic recovery plans that provide vocational training for the female workforce	• Disaster management • Finance • Labor • Medicine
Shifting rainfall and temperature patterns impair crop, livestock, and fishery yields, contributing to food insecurity	• Women suffer higher rates of anemia and nutrient deficiencies, and although women produce 60%–80% of food, less than 10% are landowners in developing countries	• Empowerment through women-centered climate-resilient farming models that encourage and assist women in gaining cultivation rights and simultaneously provide skills and training to implement resilience-building practices • Community-based reintroduction of nutrient-dense, locally available wild edibles into the regular diets • Strengthen nutritional interventions in reproductive-aged women	• Agriculture • Finance • Public health • Environment
Water insecurity and increased risk of waterborne disease	• Water scarcity forces women to walk long distances to harvest from sources that may be biologically and toxicologically contaminated, as well as increases exposure to heat and decreases time spent on other activities such as education and economic gain	• Increase accessibility to affordable home water filters • Increase public investment in water infrastructure in high-risk areas such as urban slums • Engage local female leaders and female heads of household in local, regional, and national sanitation projects to promote culturally acceptable infrastructure development that ensures women have safe and private access to hygienic facilities and clean water • Promote water-saving practices that take into account the different uses of water for women	• Technology • Public health • Environment • Finance • Urban planning
Changes in temperature, precipitation, and ecology are altering the geographic distribution of vector-borne diseases	• Pregnant women are disproportionately affected by vector-borne diseases and additionally serve the role as primary caregivers to the sick	• Collection of gender-disaggregated health data • Vector-borne surveillance systems and early-warning systems can permit effective and efficient prepositioning of resources, including bed-nets and insecticides. • Childcare facilities can support women’s care-giving role while transformation of gender norms takes place	• Public health • Research community
Climate-induced environmental change drives human migration and/or results in “trapped” populations	• Women are vulnerable to forced migration as a result of environmental change, are often excluded from migration analysis, and suffer poor health outcomes as a result of interpersonal violence and lack of reproductive healthcare; they also have fewer options in terms of migrating into economically viable situations	• Planned and well-managed migration can reduce the chance of later humanitarian emergencies, ease people out of situations of vulnerability, and capitalize on opportunities afforded to the individual by migration (e.g., moving populations away from flood zones into areas of safety and prosperity) • Proactive management of natural resources in climate “hot spots” may prevent forced migration/trapped populations • The trend of migration is from rural to urban environments—action is needed to build urban infrastructure that is sustainable and flexible to accommodate this population flux • When migrants are in refugee camps, it is essential that all possible resources are deployed to protect women’s personal safety and provide reproductive health services	• Multinational cooperation • International and domestic food organizations • Labor • Finance

Abbreviation: PM_2.5_, inhalable airborne particles with diameters that are 2.5 micrometers and smaller.

### Ensure participation

Recognizing women’s roles as educators, caregivers, holders of knowledge, and powerful agents of social change positions women to effectively design and implement culturally acceptable interventions where they are needed most. Women should be empowered as key stakeholders at the outset of any project with the understanding that combining scientific data and community knowledge will yield better results.

### Prioritize education

Education regarding the gender-specific health threats of climate change is needed within public health, policy, medicine, and general education. Additionally, investment in skills and capacity building among women will foster leadership and strengthen resilience.

### Improve data

Collecting high-quality gender-disaggregated data will enable better understanding of gender–climate–health associations and allow for predictive modeling that can inform community-based interventions and improve outcomes for both men and women.

### Enhance preplanning

A comprehensive assessment of women’s and men’s assets and vulnerabilities is foundational to any adaptation or development project, including disaster risk reduction, transportation, finance, communication, water management, technology transfer, agriculture, and health. Such assessments not only provide a more in-depth understanding of the effects of climate change but also reveal the political, physical, and socioeconomic reasons why individuals suffer disproportionately. This creates a stronger opportunity for effective intervention.

### Redefine success

Women’s health outcomes and economic prosperity can serve as surrogate markers for development, disaster risk reduction, and climate adaptation and should be used as indicators for project and policy success. Similarly, regions with poor health outcomes should be identified as “hot spots” for current and future vulnerability to climate change.

### Improve multisector coordination

Developing mechanisms for reporting and regular analysis of gender dimensions using common indicators within all sectors will increase transparency and cooperation in achieving this cross-sectoral goal.

## Conclusions

While gender has been increasingly factored into climate change projects and policy, progress has still been slow to reduce gender-based health disparities and to involve women in climate change mitigation, adaptation, and disaster risk reduction and management. The need for compliance with the monitoring processes advocated by the SDGs and the Sendai Framework are critical to address the complex interactions between poverty, gender-based social discrimination, and climate change that threaten to amplify gender-based health disparities. To support monitoring, effective mechanisms to gather and analyze data are needed. Women’s distinct social roles and potential for agency afford opportunities for promoting effective solutions to sustainability, disaster risk reduction, and solutions to health threats. High-level political engagement with the implementation of the UN landmark agreements is necessary to ensure that policies and programs move beyond traditional separations of health, gender, and environment and embrace proactive and gender-based solutions that protect women’s health.

## Abbreviations

CNS: central nervous system
SDG: Sustainable Development Goal
UNFCCC: United Nations Framework Convention on Climate Change
UNISDR: United Nations International Strategy for Disaster Reduction
